# Pharmacological inhibition of LSD1 activity blocks REST-dependent medulloblastoma cell migration

**DOI:** 10.1186/s12964-018-0275-5

**Published:** 2018-09-18

**Authors:** Keri Callegari, Shinji Maegawa, Javiera Bravo-Alegria, Vidya Gopalakrishnan

**Affiliations:** 1 Department of Pediatrics, University of Texas M.D. Anderson Cancer Center, Unit 853, 1515 Holcombe Blvd, Houston, TX 77030 USA; 2 Department of Molecular and Cellular Oncology, University of Texas M.D. Anderson Cancer Center, Houston, TX 77030 USA; 3 Department of Center for Cancer Epigenetics, University of Texas M.D. Anderson Cancer Center, Houston, TX 77030 USA; 4 Department of Brain Tumor Center, University of Texas M.D. Anderson Cancer Center, Houston, TX 77030 USA; 5 The University of Texas MD Anderson Cancer Center UTHealth Graduate School of Biomedical Sciences, Austin, USA

**Keywords:** LSD1, REST, SHH, Medulloblastoma, Tranylcypromine, HIF1A

## Abstract

**Background:**

Medulloblastoma (MB) is the most common malignant brain tumor in children. Current problems in the clinic include metastasis, recurrence, and treatment-related sequelae that highlight the need for targeted therapies. Epigenetic perturbations are an established hallmark of human MB and expression of Lysine Specific Demethylase 1 (LSD1) is elevated in MBs compared to normal tissue, suggesting that LSD1 inhibitors may have efficacy against human MB tumors.

**Methods:**

Expression of *LSD1* was examined across a publicly-available database and correlated with patient outcomes. Sonic Hedgehog (SHH) MB samples were clustered based on expression of *LSD1* and LSD1-associated *RE-1* silencing transcription factor (*REST*) target genes as well as genes involved in metastasis. Resulting clusters were examined for patient outcomes associated with *LSD1* and *REST* expression. Human SHH MB cell lines were transduced with a *REST*-transgene to create isogenic cell pairs. In vitro viability and cell migration assays were used to examine the effect of LSD1 knockdown or inhibition on these parameters.

**Results:**

We demonstrate that subsets of SHH MB tumors have elevated *LSD1* expression coincident with increased expression of its deubiquitylase, *USP7*, and *REST*. Patients with co-elevation of *USP7*, *REST*, and *LSD1* have poorer outcomes compared to those with lower expression of these genes. In SHH MB cell lines, REST elevation increased cell growth and LSD1 protein levels. Surprisingly, while genetic loss of *LSD1* reduced cell viability, pharmacological targeting of its activity using LSD1 inhibitors did not affect cell viability. However, a reduction in REST-dependent cell migration was seen in wound healing, suggesting that REST-LSD1 interaction regulates cell migration. Ingenuity pathway analyses validated these findings and identified Hypoxia Inducible Factor 1 alpha (HIF1A) as a potential target. In line with this, ectopic expression of HIF1A rescued the loss of migration seen following LSD1 inhibition.

**Conclusions:**

A subset of SHH patients display increased levels of LSD1 and REST, which is associated with poor outcomes. REST elevation in MB in conjunction with elevated LSD1 promotes MB cell migration. LSD1 inhibition blocks REST-dependent cell migration of MB cells in a HIF1A-dependent manner.

**Electronic supplementary material:**

The online version of this article (10.1186/s12964-018-0275-5) contains supplementary material, which is available to authorized users.

## Background

Medulloblastoma (MB) is an embryonal tumor that arises in the hindbrain of pediatric patients. MB is currently classified into 4 molecular subgroups: Wingless (WNT), Sonic hedgehog (SHH), Group 3, and Group 4 [1]. These subgroups are further broken down into one or more of the following subtypes: alpha (α), beta (ß), gamma (γ), or delta (δ). Patients with WNT tumors have relatively good prognosis, permitting the investigation of de-escalated treatments for these patients in clinical trials [1–4]. However, a number of patients from the other MB subgroups, especially SHH and Group 3, exhibit metastasis and recurrence [1, 3]. In fact, approximately 20% and 30% of all patients with SHH and Group 3 tumors, respectively,  present with metastasis [2, 5]. For these patients, the current standard of care is not curative and frequently results in unnecessary neurological toxicity. It is for these subsets of patients that the need for personalized and targeted therapy is dire.

Inhibitors targeting epigenetic modifiers such as histone deacetylases (HDAC), histone methyltransferases (HMT), and histone demethylases (HDM) are promising candidates in the hunt for targeted treatments. Perturbations in chromatin remodeling enzymes occur across all MB subgroups and are attractive therapeutic targets [2, 6]. Several studies have demonstrated anti-tumorigenic activity with HDAC inhibitors in MB cells and upregulation of HDAC enzymes is established to dysregulate cell cycle [7, 8]. Work in our lab has focused on studying the feasibility of targeting the *RE1*-Silencing Transcription Factor (REST), a zinc finger DNA binding protein and transcription factor, which serves as a hub for the assembly of a number of chromatin remodelers including G9a, LSD1 and HDAC1/2 [9–11]. It recruits these enzymes through its amino-terminal mSIN3A or its carboxy-terminal coREST co-repressor complexes. REST is a repressor of neurogenesis and controls the expression of a number of genes involved in neuronal differentiation [12–15]. MBs are poorly differentiated tumors and previous work from a couple of groups including ours showed aberrant REST elevation in human MBs [16–19]. Both the expression and activity of REST and coREST has been implicated in poor patient prognosis and MB progression [9, 19, 20]. Our previous work showed the feasibility of targeting REST-coREST associated G9A activity as well as of targeting REST-mSIN3A associated HDAC1/2 in human MB cell lines [19, 21]. Thus, while advances in targeting of the REST-coREST complex with HDAC and G9A inhibitors have been made, the role of another complex member, lysine specific demethylase 1 (LSD1), is poorly understood in MB [7, 22, 23]. LSD1 demethylates lysine residues 4 and 9 on histone 3 tails (H3K4/H3K9) to canonically repress transcription, although it is also known to demethylate non-histone targets such as HIF1A [24–26]. Methylation of histone H3K4/H3K9 is altered in MB, and LSD1 levels are upregulated in both neuroblastoma and MB compared to normal tissue [27, 28]. Although, LSD1 was described as a potential target in MB in 2013, its role in MB tumorigenesis has not been examined so far. In a previous study, it was demonstrated that LSD1 protein and transcript expression is increased in MB tumors over normal cerebellum, but the authors found no difference in expression between subgroups [27].

In this report, we discovered that *LSD1* gene expression is significantly elevated in the WNT, SHH, and Group 3 MB tumors compared to Group 4 MBs. This correlated with a trend for patients with metastasis to exhibit increased *LSD1* expression. Interestingly, increased *LSD1* gene expression was significantly associated with poor survival in patients with Group 3 tumors. To investigate if alterations in LSD1 activity rather than gene expression alone can be used for prognostication in SHH patients, we performed a clustering of SHH tumor samples using gene expression data of known LSD1 target genes in the brain along with target genes of its interacting partner-REST, the REST and LSD1-specific deubiquitylase (DUB) *USP7,* and genes known to contribute to MB metastasis*.* This approach identified clusters where higher *LSD1* expression, especially in the context of higher-*REST* expression, correlated with poor patient outcomes compared to patients with lower expression of the above genes. In vitro experiments with human SHH MB cell lines revealed REST elevation to contribute to increased cell growth and migration. Cell growth could be blocked by genetic knockdown of *LSD1*. Surprisingly, pharmacological inhibition of LSD1 using a panel of irreversible inhibitors did not have an effect on cell growth. However, REST-dependent migration defects were reversed by irreversible LSD1 inhibition. Ingenuity Pathway Analysis (IPA) showed genes regulating cell migration to be differentially changed following LSD1 inhibition of MB cells. Amongst these genes, *HIF1A*, a molecule known to control glioma cell migration was most significantly downregulated by LSD1 inhibition [29, 30]. Constitutive HIF1A expression restored cell migration in LSD1 inhibitor-treated REST-high MB cells. *HIF1A* expression was significantly correlated with *LSD1* and *REST* expression in SHH clusters with the metastatic α and β subgroup of patients. Thus, our data suggest that study of LSD1 inhibitors in the context of SHH α and β tumors warrants further exploration as a therapeutic option.

## Materials and methods

### Analysis of patient data

Patient data used for patient outcome and clustering analysis was sourced from previously published work done by Cavalli et al., 2017. The database is publicly available under accession number GSE85217. Z-scores for available genes were calculated and used as the basis for further examinations and correlations. Clinical data was paired with gene expression values. Two additional datasets are also publicly available on the NIH website under accession numbers GSE109401 and GSE37418.

### Statistics

Spearman correlation between gene expression profiles was calculated using GraphPad Prism 5.0 (GraphPad Software Inc., San Diego, CA, USA). All *p*-values are two-sided with a *p* < 0.05 considered to be significant. Hierarchical clustering was performed by ArrayTrack Software available at http://edkb.fda.gov/webstart/arraytrack/ using Ward’s method. p-values for comparisons between clusters based on gene expression status were obtained using the unpaired t-test with Welch’s correction.

### Cell culture

UW228 and Daoy cells were obtained from American Type Cell Culture (ATCC). Cells were cultured and maintained in Dulbecco’s modified eagle medium (DMEM) supplemented with 10% fetal bovine serum (FBS), 1× non-essential amino acids, and penicillin/streptavidin at 37 °C in a 5% CO_2_ incubator. Isogenic MB cell lines with endogenous and ectopically expressed REST (Daoy-REST; UW228-REST) were generated by viral transduction and selection for stable clones as described previously. Viral transduction of short hairpin RNA (shRNA) against *LSD1* was done using Lipofectamine 2000 (Invitrogen). Cells were selected with puromycin and expanded for further experiments.

### MTT assay

Tranylcypromine, GSKLSD1, and GSK2789552 are commercially available irreversible LSD1 inhibitors and were purchased from Sigma-Aldrich (Tranylcypromine and GSKLSD1) and Chemitek (GSK2789552). MTT assays were carried out according to standard protocol. Cells were seeded at a density of 3500 cells/well and incubated overnight. The following day, media was removed and fresh media with a range of drug doses were added to cells. At the end of 24, 48, and 72 h following drug addition, MTT reagent was added and spectrophotometer readings were taken at 595 nm and 650 nm.

### Quantitative reverse transcriptase real-time polymerase chain reaction (qRT-PCR)

RNA was isolated from cells using Qiagen RNA mini-prep kits. cDNA was prepared from isolated RNA using iSCRIPT and qRT–PCR reactions were performed following standard protocol in 96-well plates using a Roche LightCycler 96 machine. Cycle amplification data was processed with LightCycler software. Relative mRNA expression was calculated using the comparative ΔΔ-Ct (cycle threshold) method and was normalized using 18S expression.

Primers used are as follows:

- *REST forward: 5’-GTAGGAGCAGAAGAGGCAGAT-3’*
- *REST reverse: 5’-GCTTCACGTTCTTCTACTGCT-3’*
- *18S forward*: *5’-CGGCGACGACCCATTCGAAC-3’*
- *18S reverse*: *5’-GAATCGAACCCTGATTCCCCGTC-3’*
- *BMP2 forward*: *5’-AACACTGTGCGCAGCTTC-3’*
- *BMP2 reverse: 5’-TTCGGTGATGGAAACTGCTA-3’*
- *HIF1A forward*: *5’-TGCTCATCAGTTGCCACTTC-3’*
- *HIF1A reverse*: *5’-TCCTCACACGCAAATAGCTG-3’*

### Boyden chamber assay

24-well transwell Matrigel migration assay plates and control inserts were purchased from Corning (Catalog #354480). Briefly, approximately 2.5 × 10^4^ cells were seeded in 8 μM pore-size transwell chambers. The bottom chamber was filled with 20% FBS to act as a chemoattractant. Cells were allowed to incubate for 22 h and were then fixed, stained with Hemistat triple pack and imaged under a microscope. Cell nuclei were counted from 4× images taken of the membrane insert. CellCounter software was used to automate the counting process. Cells were plated in triplicate and averaged.

### Scratch wound healing assay

Cells were seeded onto 6-well plates in equal number and allowed to rest until confluent. A wound of equal width was made with a sterile 10 μL pipette tip along the middle of the well. Media was aspirated and replaced with media containing 75 uM GSKLSD1 or DMSO. Time points were taken every 6 h until the wound closed. Cells were transfected with a pcDNA3 plasmid expressing a constitutively active mutant *HIF1A* with P402A and P564A substitutions (Addgene) [31].

### RNA-Seq analyses

Daoy cells were grown in 100 cm^2^ plates and treated with either 75 μM of GSKLSD1 or DMSO. Cells were washed with phosphate buffered saline (PBS), pelleted, and snap frozen to be stored at − 80 °C. RNA Seq was performed at Active Motif (Carlsbad, CA) and data was processed into GeneDiff excel files. Data was examined by IPA for function assessment of gene expression changes.

## Results

### Analysis of patient samples based on LSD1 expression and activity

**Fig. 1:**
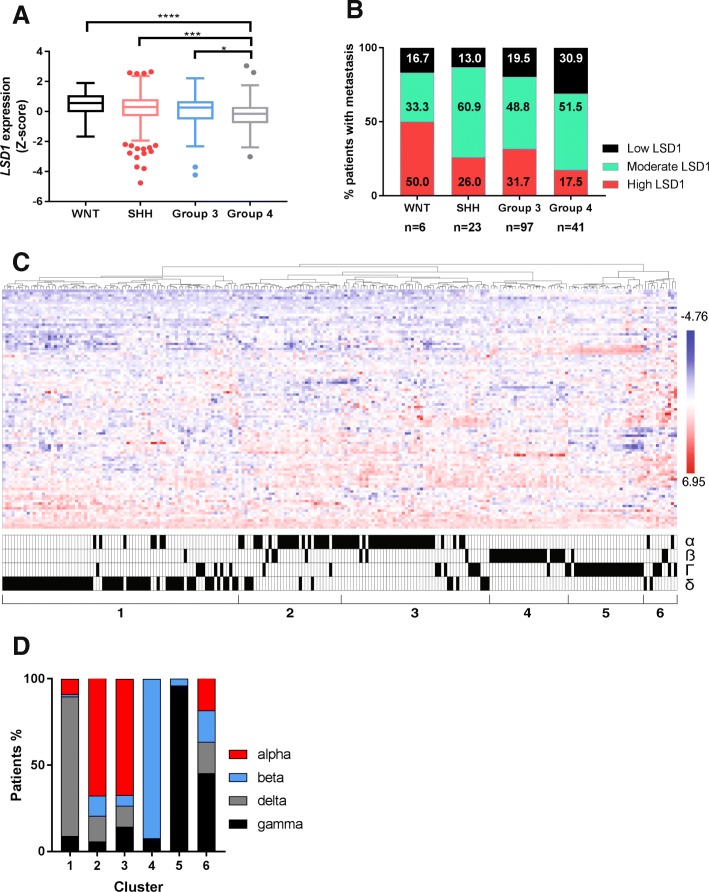
Analysis of medulloblastoma patient samples by LSD1 expression and activity. Analysis of publicly available dataset containing 763 MB patient samples (GSE85217). **a**, Tukey box plots of Z-scores of *LSD1* gene expression. The data points (circles) outside of the boxes represent outliers. (*p < 0.01; ***p < 0.003; ****p < 0.0001). **b**, Examination of patients with metastasis divided by *LSD1* expression across subgroups. **c**, Clustering assay of SHH MB patient samples identified 6 distinct clusters based on predicted LSD1 activity. These clusters contained subtype–specific information (α, ß, δ, or Γ) provided in the bottom panel. **d**, Breakdown of SHH MB patient subtypes (alpha, beta, delta, gamma) per cluster (Cluster 1 *n* = 78; Cluster 2 *n* = 34; Cluster 3 *n* = 49; Cluster 4 *n* = 26; Cluster 5 *n* = 25; Cluster 6 *n* = 11)

To investigate subgroup-specific differences in *LSD1* gene expression, we examined a publicly available dataset (GSE85217) of 763 MB tumor samples. A significant analysis of variance (ANOVA) in *LSD1* gene expression was observed (*p* < 0.0001; Fig. 1a) [3]. Within this dataset, Group 4 MB samples had significantly lower average *LSD1* gene expression compared to WNT, SHH, or Group 3 tumors (Fig. 1a).

To better understand how LSD1 levels could affect patient outcomes, we divided patient samples based on the average Z-score of their *LSD1* expression into three groups: low (Z-score < − 0.40), moderate (− 0.40 < Z-score < 0.40), and high (Z-score > 0.40). Mantel-Cox analysis of survival only revealed a significant difference between low and moderate *LSD1* across all of the MB patients (*p* < 0.05; Additional file 1A). A significant difference of survival was noted in patients with Group 3 but not the other MB subgroups (Additional file 1B-E). Although previous studies have shown that histone H3K4 methylation is perturbed in SHH MB, we did not see significant differences in survival based on *LSD1* expression alone [28]. However, the SHH subgroup did have the most variable *LSD1* expression range with outliers on both the low and high spectrum (Fig. 1a). Using the same classification, we observed that the majority of patients with metastasis across all MB subgroups had high or moderate levels of *LSD1* (Fig. 1b). These results suggest that *LSD1* gene expression alone may be predictive of metastasis but not survival in SHH MB patients.

Additional analysis of two other smaller MB datasets revealed a positive correlation of *LSD1* and coREST-associated *REST* across SHH MB samples. However, these datasets did not include enough data or patient outcomes for further analysis (Additional file 1F, G; *r* = 0.48 *p* = 0.08; *r* = 0.92, *p* < 0.05).

To ask if *LSD1* co-elevation with other genes or perturbation of *LSD1* activity in SHH tumors may have more predictive value, we performed clustering analysis using a cohort of 91 LSD1 target genes, the deubiquitylase *USP7*, and 8 REST and metastasis related genes in the brain [27, 32, 33] (Additional file 2A). This identified 6 clusters that aligned with subsets of the SHH subtypes (alpha (α), beta (ß), gamma (γ), delta (δ) published previously by Cavalli et al. (Fig. 1c) (3). The composition of the clusters was as follows: Cluster 1–80% of SHH δ tumors, Clusters 2 and 3 together added up of 78% SHH α tumors, Cluster 4–92% SHH ß tumors, Cluster 5–96% SHH γ tumors, and Cluster 6 was more undefined (Fig. 1d).

### Clustering of patient samples based on LSD1 activity is correlated with patient outcomes

**Fig. 2:**
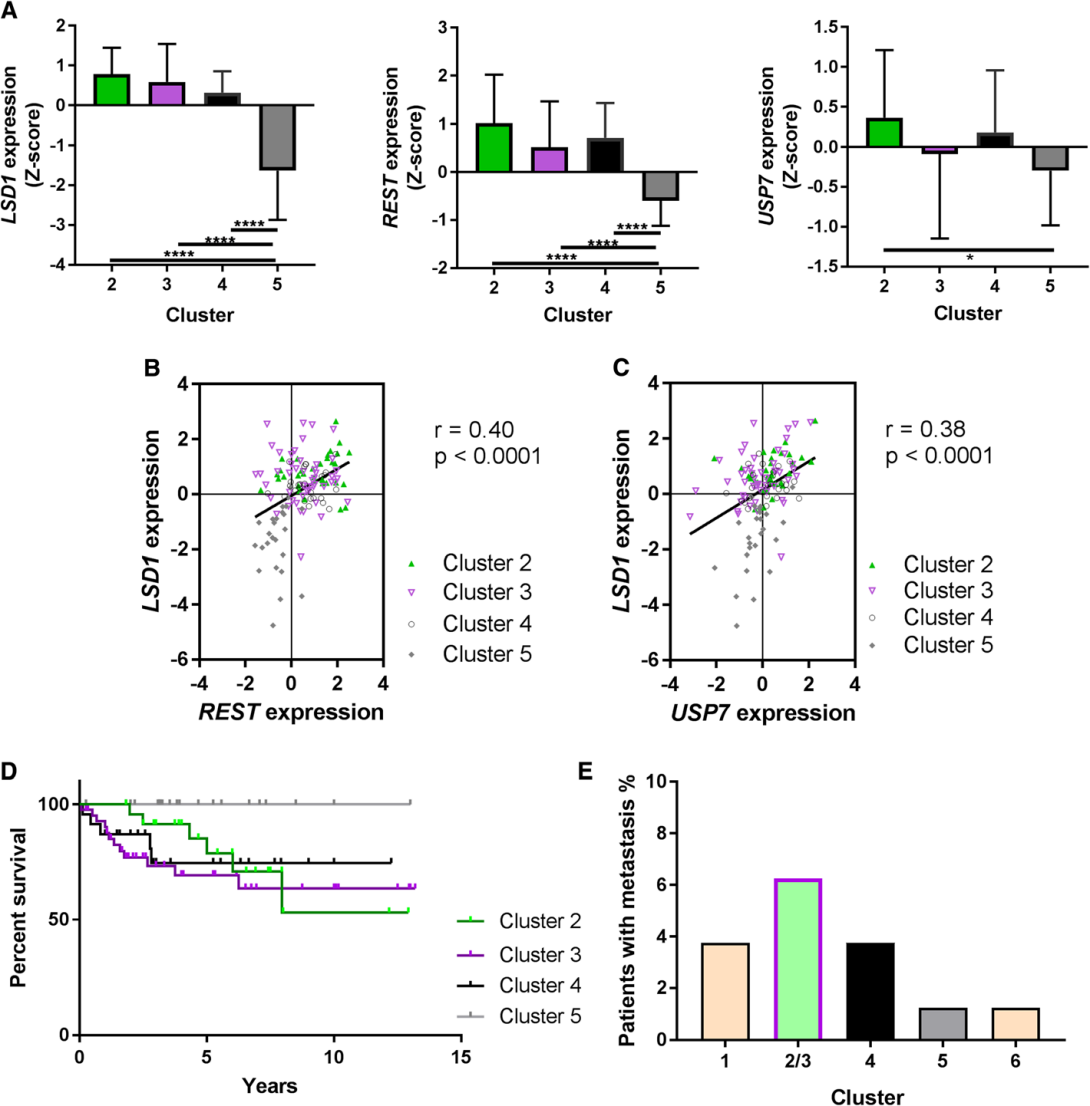
MB patient samples clustered based on LSD1 activity are related to patient outcomes. **a**, Bar graphs of expression of *LSD1, REST,* and *USP7* in Clusters 2, 3, 4, and 5 (*p < 0.05; ****p < 0.0001). Combined expression of *USP7* with Cluster 2, 3, and 4 was significantly higher than Cluster 5 (*p* < 0.05). **b**, Scatter plot of correlation of *REST* and *LSD1* across Clusters 2–5 (*r* = 0.40, *p* < 0.0001). **c**, Scatter plot of correlation of *LSD1* and *USP7* across Clusters 2–5 (*r* = 0.38, *p* < 0.0001). **d**, Patient survival (Kaplan-Meier curves) associated with Clusters 2, 3, 4, and 5. Clusters 2 vs. Cluster 5 had a significant Mantel-Cox test (*p* < 0.05). Cluster 3 vs. Cluster 5 had a significant Mantel-Cox (*p* < 0.01) and Gehan-Breslow-Wilcoxon test (*p* < 0.05). Cluster 4 vs. Cluster 5 had a significant Mantel-Cox (*p* < 0.01) and Gehan-Breslow-Wilcoxon test (*p* < 0.05). Combined Clusters 2 and 3 vs. Cluster 5 had a significant Mantel-Cox (*p* < 0.05) and Gehan-Breslow-Wilcoxon test(*p* < 0.05). ctrl: control. **e**, Percentage of metastasis within each cluster

Using the associated gene expression microarray data, we examined the expression of *REST*, *LSD1* and *USP7* in the 6 clusters generated above (Additional file 2B). Interestingly, we found that the clusters containing the majority of the pediatric brain tumors (Clusters 2/3, 4, and 5; α, ß, and Γ respectively) had significant differences in the expression patterns of *LSD1, REST,* and *USP7* (Additional file 2B)*.* Focusing on these clusters, we compared Clusters 2–5 using bar plots. Cluster 2 (subset of SHH α tumors) was characterized by the highest increases in *LSD1*, *REST*, and *USP7* expression, while Cluster 5 (SHH γ tumors) exhibited the opposite pattern, with low expression of these genes (Fig. 2a). Clusters 3 (subset of SHH α tumors) and 4 (SHH ß) exhibited *LSD1* and *REST* expression comparable to that in Cluster 2, but lacked significantly higher expression of *USP7*. In line with this, we also observed a strong correlation between *LSD1* and *REST* (Fig. 2b; *r* = 0.40, *p* < 0.0001) and *LSD1* and *USP7* (Fig. 2c; *r* = 0.38, p < 0.0001). Of note, an overall significant positive correlation between *LSD1* and *REST* and *LSD1* and *USP7* was also noted within all SHH MBs (Additional file 2C, D; *r* = 0.20, *p* = 0.002, *r* = 0.39. p < 0.0001). Further, tumors in Clusters 1 and 6 had high *LSD1* but not *REST* or *USP7* expression levels (Additional file 2B). These results indicate that co-elevation of *REST* and *LSD1* transcript or a simultaneous increase in their activity could occur in subsets of SHH tumors.

Examination of patient outcomes associated with these clusters revealed a significant difference in Mantel-Cox analysis of survival curves of Clusters 2–4 versus Cluster 5 (Fig. 2d; Additional file 2E; *p* < 0.05). Analysis of metastasis occurrence within these clusters showed that Clusters 2/3 and 4 contained a higher percentage of patients with metastasis than Cluster 5 (Fig. 2e). Together, these results suggest that subsets of patients within the SHH α and ß MB type have increased *LSD1* and *REST* levels or activity and exhibit poor outcomes.

### *LSD1* is required for MB cell viability

**Fig. 3:**
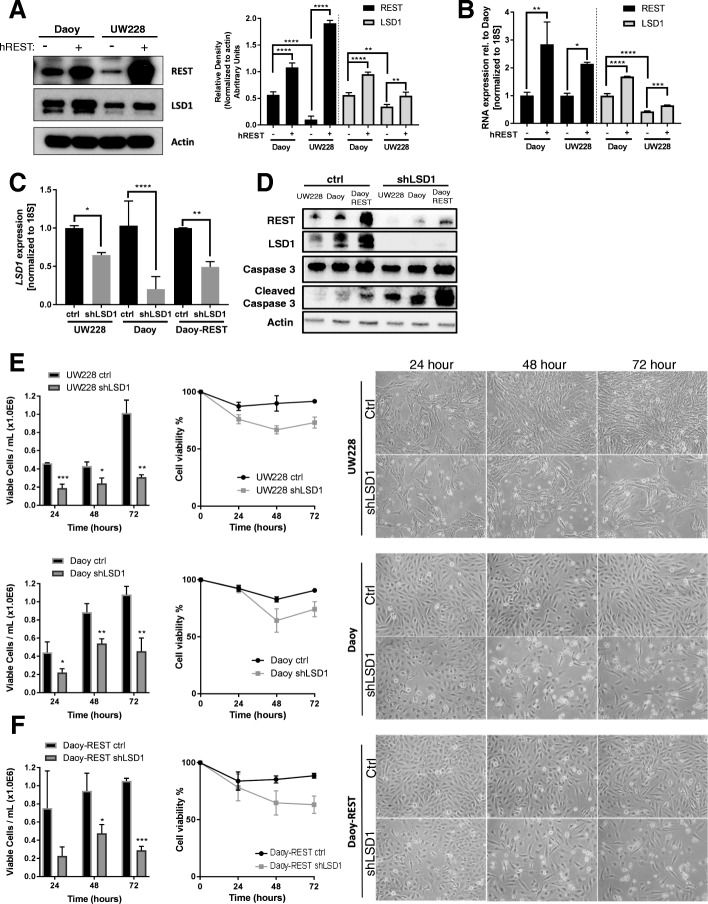
LSD1 expression is increased by elevated REST and cell growth is blocked by LSD1 knockdown in SHH MB cell lines. **a**, Western blot of REST and LSD1 protein levels in cell pairs (UW228, Daoy) with and without the human *REST* transgene (hREST; UW228-REST, Daoy-REST) (left panel). Quantification of REST (black) and LSD1 (grey) protein density normalized to actin loading control (right panel; **p < 0.01, ****p < 0.000). **b**, qPCR analysis of *REST* (black) and *LSD1* (grey) expression normalized to *18S* (*p < 0.05, **p < 0.01, ***p < 0.001, ****p < 0.0001). **c**, qPCR analysis of *LSD1* expression in UW228, Daoy, and Daoy-REST cells treated with shLSD1 (*p < 0.05, **p < 0.01, ****p < 0.0001). **d**, Western blot of REST, LSD1, Caspase 3, Cleaved Caspase 3, and actin protein levels in control (ctrl) and shLSD1 treated cells. **e**, Cell viability as measured by trypan blue counting in UW228, Daoy, and **f**, Daoy-REST cells. Graphs represented as viable cell count/mL and % cell viability. Pictures represent cell confluency at 24, 48, and 72 h post-transduction (*p < 0.05; **p < 0.01;***p < 0.001)

To understand the role of *REST* and *LSD1* in MB cells, we first measured LSD1 and REST proteins levels in Daoy and UW228 SHH-MB cell lines. As expected, UW228 cells had lower REST and LSD1 when compared to DAOY cells (Fig. 3a). These cells were then engineered to ectopically express human *REST* transgene (Daoy-REST; UW228-REST) and cell extracts were evaluated by Western blotting for levels of REST and LSD1. We observed a significant increase in REST levels, which coincided with an increase in LSD1 protein levels (Fig. 3a). qPCR analysis of Daoy and UW228 cells also demonstrated a significant upregulation of *REST* transcript (3-fold and 2-fold) in high-REST cells (Fig. 3b). Surprisingly, *LSD1* transcript showed a small but significant REST-associated elevation as well (1.5-fold and 1.3-fold), relative to their expression in parental cells (Fig. 3b).

To determine the relevance of LSD1 for growth of REST-expressing MB cells, *LSD1* was knocked down in UW228, DAOY and Daoy-REST cells and its effect on cell numbers was determined. Efficiency of *LSD1* knock down was confirmed by qRT-PCR and Western blot analyses (Figs. 3c, d). Interestingly, *LSD1* loss promoted a reduction in REST protein levels in all three cell lines (Fig. 3d). LSD1 loss in Daoy and UW228 cells in response to *LSD1*-specific shRNA (*shLSD1*) expression caused a significant reduction in their growth as measured by MTT assays when compared to cells transduced with control (Ctrl) *shRNAs* (Fig. 3e; Additional file 3A-C). A reduction in the number of MB cells was also noted in light microscopy images following *LSD1* knockdown when compared to control *shRNA* transduced cells (Fig. 3e and f, right panels; Additional file 3A-C). As expected, REST elevation caused an early increase in cell growth of Daoy cells, but this cell growth advantage was reduced by genetic knockdown of *LSD1* (Fig. 3f; Additional file 3A-C). These results indicate that LSD1 is required for REST-dependent growth of MB cells. *LSD1* knockdown also promoted Caspase-3 cleavage, indicating induction of apoptosis (Fig. 3d).

### Pharmacological inhibition of LSD1 counters REST-dependent MB cell migration

To validate the above findings, we also examined the effect of pharmacological inhibition of LSD1 activity on MB cell viability. A panel of irreversible LSD1 inhibitors (Tranylcypromine, GSKLSD1 and GSK2789552) was evaluated with MTT assays using isogenic pairs of UW228/UW228-REST and Daoy/Daoy-REST cells [34]. To our surprise, we observed that none of the irreversible inhibitors had any effect on the growth or number of SHH MB cell lines UW228 and Daoy cells up to a 100 μM dose (Additional file 4A). REST elevation did not influence the effect of these agents on UW228-REST or Daoy-REST cells (Additional file 4B).

**Fig. 4:**
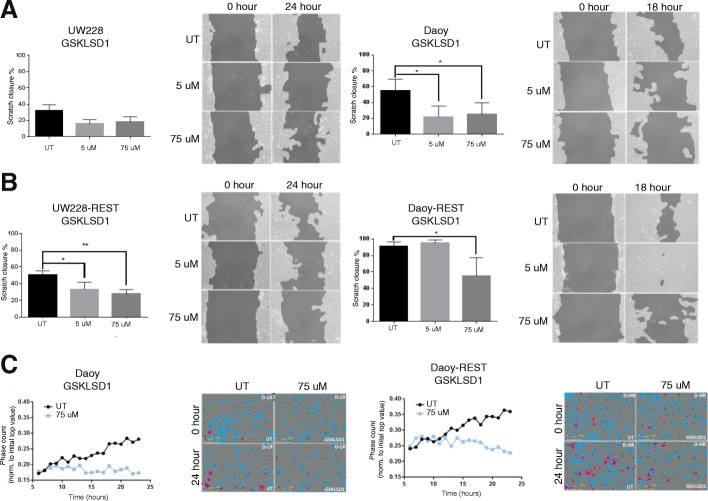
Pharmacological inhibition of LSD1 reduces cell migration of SHH MB cell lines. **a**, Scratch wound migration assay of UW228 and Daoy cells at low dose (5 uM) and high dose (75 uM) of GSKLSD1. **b**, Scratch wound migration assay of UW228-REST and Daoy-REST cells at low dose (5 uM) and high dose (75 uM) of GSKLSD1 (*p < 0.05, **p < 0.01). UW228-REST and Daoy-REST cells migrate twice as fast as UW228 and Daoy cells, respectively (*p* < 0.05). **c**, Transwell migration of Daoy and Daoy-REST cells over 24 h at 75 uM of GSKLSD1. Slope of migration is significantly reduced (*p* < 0.05)

However, we observed a REST-dependent increase in migration of MB cells in vitro. Daoy cells with higher *REST* expression could more rapidly narrow the wound in scratch assays (18 h) compared to UW228 cells, which required 24 h for a similar effect (Fig. 4a, B). This effect of *REST* elevation on cell migration was further corroborated by similar studies comparing migration of isogenic Daoy and Daoy-REST as well as UW228 and UW228-REST cell pairs (Fig. 4a, b). Interestingly, Daoy-REST cells displayed resistance to LSD1 inhibition at the lower dose (5 μM), requiring a dose of 75 μM to significantly reduce migration (Fig. 4b). These results were confirmed by Boyden chamber assays to determine transwell migration of Daoy and Daoy-REST cells to fetal bovine serum (FBS). GSKLSD1 treatment at 75 μM was sufficient to significantly reduce cell migration over time in both Daoy and Daoy-REST cells (Fig. 4c). Together our results suggest that irreversible LSD1 inhibition blocks REST-dependent MB cell migration.

### Irreversible inhibition of LSD1 alters cellular migration machinery

**Fig. 5:**
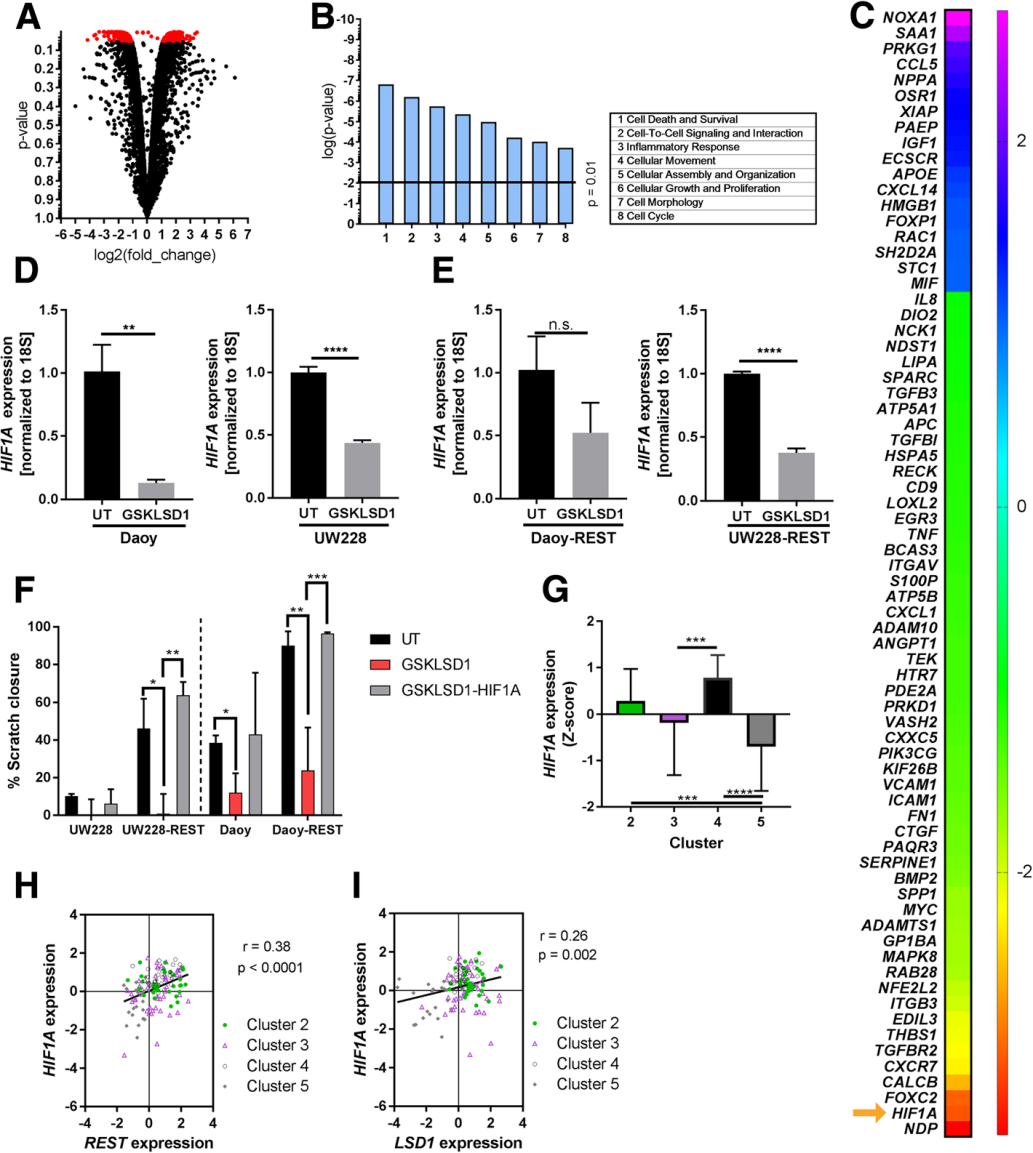
Pharmacological inhibition of LSD1 alters expression of MB and migration related genes. **a**, Volcano plot of expression changes from RNA-sequencing data. 451 altered genes with *p* < 0.05. **b**, Ingenuity pathway analysis of function revealed top functions outside of cancer. **c**, Heat map of log2fold gene expression changes in the cell adhesion and migration function. Orange arrow highlights *HIF1A*. **d,** qPCR analysis of *HIF1A* expression in Daoy and UW228 cells treated with GSKLSD1 (**p < 0.01; ****p < 0.0001). **e,** qPCR analysis of *HIF1A* expression in Daoy-REST and UW228-REST cells treated with GSKLSD1 (*p* = 0.07; ****p < 0.0001). **f**, Scratch wound migration assay of UW228, UW228-REST, Daoy, and Daoy-REST cells in three conditions: untreated (UT), GSKLSD1 (75 uM), or GSKLSD1-HIF1A (75 uM) (*p < 0.05, **p < 0.01, ***p < 0.001). **g**, Bar plot of *HIF1A* expression in Clusters 2, 3, 4, and 5 (***p < 0.001; ****p < 0.0001). **h**, Scatter plot of correlation of *REST* and *HIF1A* across Clusters 2, 3, 4, and 5 (*r* = 0.36, *p* < 0.001). **I**, Scatter plot of correlation of *LSD1* and *HIF1A* across Clusters 2, 3, 4, and 5 (*r* = 0.27, *p* < 0.01)

To study the molecular changes in MB cells in response to drug treatment, we performed RNA-Seq analyses of Daoy cells treated with 75 μM of GSKLSD1. IPA was performed to identify changes in the transcriptome. Volcano plot represents a total of 451 genes that were significantly altered in response to drug treatment (Fig. 5a). Of these, 200 genes were upregulated and 251 were downregulated by GSKLSD1 treatment (*p* < 0.05; Fig. 5a). Functional analysis identified several altered functions relevant to cancer (Fig. 5b). Gene expression changes controlling cell survival/death, cell-cell interaction and cellular movement were a few of the most highly altered molecules (Fig. 5b). Since LSD1 and REST elevation was associated with a higher incidence of metastasis in patients (Fig. 1), we carried out a more detailed examination of the “cellular adhesion and movement” category, which identified a number of significantly altered genes related to tumor cell migration (Fig. 5c). Interestingly, one of the most significantly reduced genes in this subset was hypoxia inducible factor 1 alpha (*HIF1A*).

Work from other groups has implicated REST and LSD1 in the control of *HIF1A* expression and activity [26, 35–37]. Consistent with these findings, GSKLSD1 significantly downregulated *HIF1A* levels in Daoy and UW228 cells (Fig. 5d; *p* < 0.01 and *p* < 0.0001). Further, REST elevation countered reduction in *HIF1A* in Daoy-REST cells, but not in UW228-REST cells (Fig. 5e; *p* < 0.0001 and *p* > 0.05).

To verify the role of HIF1A in GSKLSD1-associated migration blockade, MB cell lines were transfected with a plasmid expressing a constitutively active mutant of *HIF1A*, treated with the GSKLSD1 (75 μM), and effect on cell migration was examined by scratch wound assay. A significant rescue of wound closure was observed in Daoy-REST and UW228-REST cells, but not Daoy and UW228 cells, treated with GSKLSD1 and *HIF1A* (Fig. 5f, *p* < 0.01). These results implicate *HIF1A* in promoting LSD1-dependent cell migration in REST-high MB cells.

Analysis of MB patient samples revealed a significant elevation of HIFA in Clusters 2 and 4 compared to Cluster 5 (Fig. 5g). A positive correlation between *REST* and *HIF1A* expression was also seen across the clusters that were predominantly comprised of pediatric MB tumors, and across all SHH MBs (Fig. 5h; *r* = 0.38; p < 0.0001; Additional file 5A, B). Further, *HIF1A* expression was highest in tumors with the largest increases in *REST* expression (Fig. 5i; Clusters 2 and 4). *LSD1* and *HIF1A* expression were are also positively correlated in these samples (Fig. 5i; *r* = 0.26; *p* < 0.01). These correlations were also recapitulated in two smaller patient cohorts (Additional file 5A-D). Together, our results indicate that REST-associated LSD1-activity regulates *HIF1A* expression and that *HIF1A* may contribute to REST-LSD1 dependent MB cell migration.

## Discussion

LSD1, also called KDM1A/AOF2/BHC110, is a key component of several transcriptional repressor complexes, where it mediates demethylation of histone H3K4 mono and dimethyl marks, and turns off gene expression [38–40]. It is also known to activate transcription by demethylation of the repressive histone H3K9 mono and dimethyl marks [41]. Of importance to this study is the association of LSD1 with the REST-coREST complex, which controls neuronal lineage specification in neural cells [12, 42, 43]. LSD1 upregulation has been described in MB tumors by a previous study which examined its gene and protein expression across the different MB groups in a cohort of 93 patient samples [27]. However, this study did not find a significant difference in *LSD1* gene expression between the MB groups, although a separate study by Dubuc et al., demonstrated significant perturbations in histone H3K4 methylation, especially across Group 3 and Group 4 MB samples [28, 44]. In this study, we analyzed a larger, publicly available patient dataset of 763 MB samples, and found by contrast a significant increase in *LSD1* expression in SHH, Group 3, and Group 4 compared to WNT MB samples. Importantly, *LSD1* expression appeared to be elevated in a majority of patients with SHH, Group 3, and Group 4 tumors who exhibited metastasis, suggesting that targeting LSD1 may have relevance to patients with metastatic disease.

In the current report, we quantified LSD1 activity across patient samples from the SHH MB subgroup, where survival did not seem to correlate with changes in *LSD1* transcript expression alone. LSD1 activity and or stability is known to be regulated at the post-transcriptional level either by its phosphorylation, hydroxylation or its association with transcription factors such as REST [9, 24, 25, 43]. To examine SHH MB samples based on LSD1 function specifically in the context of REST transcriptional activity, we clustered samples based on expression of known LSD1 targets along with known REST-target genes and *USP7*, a gene that encodes a deubiquitylase controlling REST and LSD1 protein stability. Thus, six distinct clusters were generated, which helped identify co-elevation of REST, LSD1 and USP7 in a subset SHH α tumors (Cluster 2), thus potentially correlating REST-LSD1 protein stability to metastasis [45, 46]. While Cluster 3 (the second subset of SHH α tumors) also had elevated *REST* and *LSD1* gene expression, *USP7* expression was not as elevated and consistent with this, LSD1 activity was not as elevated. SHH α tumors are also characterized by *MYCN* expression and p53 mutations in non-infant children with a median age of 8 years [3]. Although, this has not been evaluated in this study, it is possible that LSD1 (and REST) dysregulation could contribute to the poor prognosis in this cluster of patients by affecting p53 and MYCN function in the tumor [47]. Cluster 4 (SHH ß subtype) tumors, which occur predominantly in infants, was also associated with increased *LSD1* and *USP7* expression, but had a more modest increase in *REST* gene expression. Thus, the poor prognosis seen with this cohort of patients may be either less-REST dependent, or more dependent on REST protein levels, which is controlled by β-TRCP complex of ubiquitin ligases, and DUBs such as USP15 and USP7 [48]. Clearly, Cluster 5 tumors with a pattern of low *REST*, *LSD1* and *USP7* gene expression included patients with SHH γ patients, who are known to have good prognosis [3]. Finally, Cluster 1 included samples from patients with SHH δ subtypes of MBs. Here, a dramatic reduction in overall survival correlated with elevated *LSD1* expression in the absence of upregulation of *REST* expression. This is interesting, as SHH δ patients are largely adults with a median age of 26 years [3]. Adult MB is rare and treatment protocols are identical to that used for pediatric patients, despite differences in underlying tumor biology [49, 50]. Many of these patients present with telomerase reverse transcriptase (*TERT*) promoter mutations or methylation, increasing the expression of *TERT* to contribute to oncogenesis [51–53]. Interestingly, REST and LSD1 have both been implicated in repressing *TERT* gene expression by promoting repressive epigenetic marks to promote chromatin condensation [54, 55]. Whether, LSD1 elevation, dependent or independent of REST activity, affects *TERT* expression remains to be evaluated in laboratory studies. Thus, this analysis is the first to identified subsets of patients who may have increased activity of REST-LSD1 complex or LSD1 activity in the context of other transcription factors, and who may benefit from treatment with LSD1 inhibitors [3, 27].

Consistent with our findings from analyses of patient data, our in vitro experiments confirmed that elevation of *REST* expression increased MB cell growth and migration. This elevation in *REST* occurred concomitant with increased *LSD1* expression at both the transcript and protein levels. Changes in protein levels can be partly explained by the fact that the stability of both REST and LSD1 stability is controlled by USP7 [45]. However, other components of the REST-coREST complex such as G9a are also increased in a REST-dependent manner. A potential explanation may be that increases in REST protein levels may cause sequestration of other complex components. Indeed, the LSD1-RCOR1 interaction protects LSD1 from proteasomal degradation [34, 38]. The decrease in REST levels associated with LSD1 loss in our studies suggests that the reverse may also be true. However, the increase in LSD1 levels was higher in Daoy-REST cells than in UW228-REST cells, although higher levels of exogenously expressed REST could be maintained in UW228-REST cells. Thus, there probably exists additional explanations for how stoichiometry of the REST complex is preserved to allow greater activity. The reason for co-elevation of REST and LSD1 transcript is not clear at this time.

The observation that LSD1 knockdown but not pharmacological inhibition of LSD1 suppressed growth of SHH MB is intriguing. LSD1 is known to act as a scaffold to allow maintenance of coREST complex integrity [43]. Thus, it is conceivable that loss of LSD1 could destabilize the REST-coREST complex, which is supported by the observation that REST levels decline upon LSD1 knockdown. Since REST is required for MB cell viability, loss of LSD1 and REST would be expected to affect cell survival. GSKLSD1 is an irreversible LSD1 inhibitor derived from tranylcypromine and made more specific to LSD1 by the addition of a N-amine substituent [56–58]. This class of inhibitors blocks LSD1 demethylase activity by blocking FAD-dependent cleavage of mono- and di- methyl residues. However, LSD1 inhibition may not necessarily affect REST-coREST stability. In that case, LSD1 may retain its scaffolding function and REST activity may only be partially inhibited. This possibility remains to be investigated in future studies.

Although pharmacological inhibition of LSD1 with irreversible inhibition did not affect cell growth, cell migration was affected by GSKLSD1. Further, we found that REST-dependent *HIF1A* upregulation was reduced by irreversible LSD1 inhibition, suggesting that the REST-LSD1 complex may control *HIF1A* gene expression and/or function. Whether post-transcriptional mechanisms of regulation of HIF1A also occurs in our system, remains to be investigated. The contribution of HIF1A to the control of cell migration has been reported in a number of tumors including gliomas, and our findings are consistent with these observations [29, 30]. We observed that ectopic expression of constitutively active HIF1A rescued the loss of cell migration seen following GSKLSD1 treatment of REST-high MB cells. Together, our results suggest a subtype-specific relevance for LSD1 inhibitors as a therapeutic option and identify HIF1A as a novel downstream target of irreversible LSD1 inhibition in the context of REST elevation.

## Conclusions

Our work demonstrates that examination of *LSD1* transcript expression is an insufficient measure to assess the contribution of *LSD1* to MB tumors. Further, we show that LSD1 activity may be perturbed in the context of REST protein dysregulation in SHH α and ß tumors. Surprisingly, a strong correlation between *LSD1* elevation and poor survival was noted in patients with SHH-δ tumors, which exhibit deregulated *TERT* expression. These results suggest that specific subsets of patients with SHH tumors may benefit from treatment with LSD1 inhibitors. Our in vitro assays and IPA data appear to support a role for LSD1 in metastatic MBs and identify HIF1A as a novel potential target of LSD1 inhibition in the context of REST. However, additional studies are needed to fully understand the extent of LSD1 regulation of MB tumor biology.

## Additional files

Additional file 1: **A-E,** Patient samples were stratified into low (*n* = 207), moderate (*n* = 210), and high (*n* = 213) *LSD1* expression based on Z-score expression and survival curves were compared. **A,** Across all MBs, high and low *LSD1* expression group survival curves were insignificant in Gehan-Breslow-Wilcoxon test of early survival (*p* = 0.10) but moderate and low *LSD1* expression survival curves were significant in Mantel-Cox test (*p* < 0.05). **B-E,** Across the MB subgroups, only Group 3 samples had significantly difference curves. Overall survival data from Group 3 tumors related to *LSD1* expression. High vs. low *LSD1* had significant Gehan-Breslow-Wilcoxon and Mantel-Cox tests (p < 0.05). Moderate vs. low *LSD1* had a significant Gehan-Breslow-Wilcoxon test (*p* = 0.06). Moderate vs. high *LSD1* was insignificant. **F,** Scatter plot of *REST* and *LSD1* transcript correlation in SHH MB patients from dataset GSE37418 (*n* = 10; *r* = 0.48; *p* = 0.08). **G,** Scatter plot of *REST* and *LSD1* transcript correlation in SHH MB patients from dataset GSE109401 (*n* = 5; *r* = 0.92; *p* < 0.05). (PDF 987 kb)

Additional file 2: **A,** Listed genes used for clustering analysis in the order of top-down pictured in Fig. 1C. **B,** Box plots of *LSD1, REST,* and *USP7* expression (*p < 0.05;**p < 0.01; ***p < 0.001;****p < 0.0001). **C,** Scatter plots of *REST* and *LSD1* correlation across the whole SHH MB cohort and across each Cluster 1–6 (Cluster 1 *n* = 78; Cluster 2 *n* = 34; Cluster 3 *n* = 49; Cluster 4 *n* = 26; Cluster 5 *n* = 25; Cluster 6 *n* = 11). **D,** Scatter plots of *USP7* and *LSD1* correlation across the whole cohort and across each Cluster 1–6. **E,** Kaplan-Meier survival curve of all Clusters 1–6. (PDF 1450 kb)

Additional file 3: Light microscopy images of Daoy, UW228, and Daoy-REST cells treated with control and shLSD1 at **A,** 24 h, **B,** 48 h, and **C,** 72 h. Experiments were completed in triplicate. (PDF 14333 kb)

Additional file 4: **A,** MTT assay of 24 h and 48 h time points with three different LSD1 inhibitors (Tranylcypromine, GSKLSD1, and GSK2789552) in Daoy and UW228 cells showing no dose-response in response to up to 100 uM drug dosage. **B,** MTT assay of 24 h and 48 h timepoints with three different LSD1 inhibitors (Tranylcypromine, GSKLSD1, and GSK2789552) in isogenic high-REST counterparts, Daoy-REST and UW228-REST cells, showing no dose-response in response to up to 100 uM drug dosage. (PDF 1067 kb)

Additional file 5: **A,** Scatter plots of *REST* and *HIF1A* correlation across the whole SHH MB cohort and across each Cluster 1–6. Clusters 3 and 4 had majority of points located in the top right quadrant of the graph, indicating high expression of these transcripts, while Cluster 5 had lower left localization indicating lower expression levels. **B,** Scatter plots of *REST* and *HIF1A* transcript correlation in SHH MB patients from dataset GSE37418 (*n* = 10; *r* = 0.59; *p* < 0.05). G, Scatter plot of *REST* and *HIF1A* transcript correlation in SHH MB patients from dataset GSE109401 (*n* = 5; *r* = 0.42; *p* = 0.23). **. C,** Scatter plots of *LSD1* and *HIF1A* correlation across the whole SHH MB cohort and across each Cluster 1–6. Clusters 2–4 had majority of points located in the top right quadrant of the graph, indicating high expression of these transcripts, while Cluster 5 had lower left localization indicating lower expression levels. **D,** Scatter plots of *LSD1* and *HIF1A* transcript correlation in SHH MB patients from dataset GSE37418 (n = 10; *r* = 0.22; *p* = 0.27). G, Scatter plot of *REST* and *HIF1A* transcript correlation in SHH MB patients from dataset GSE109401 (n = 5; *r* = 0.67; *p* = 0.11). (Cluster 1 *n* = 78; Cluster 2 *n* = 34; Cluster 3 *n* = 49; Cluster 4 *n* = 26; Cluster 5 *n* = 25; Cluster 6 *n* = 11). (PDF 1382 kb)

## Abbreviations

BMP2: Bone Morphogenic Protein 2
coREST: Corepressor REST
HIF1A: Hypoxia Inducible Factor 1 alpha
LSD1: Lysine Specific Demethylase 1
MB: Medulloblastoma
REST: RE1 Silencing Transcription Factor
SHH: Sonic Hedghog
USP7: ubiquitin specific protease 7
WNT: Wingless
