# Supplementary figures and images for: Pharmacological inhibition of LSD1 activity blocks REST-dependent medulloblastoma cell migration
Source: Cell Commun Signal. 2018 Sep 18;16:60. doi: 10.1186/s12964-018-0275-5 (PMC6145331; doi:10.1186/s12964-018-0275-5)

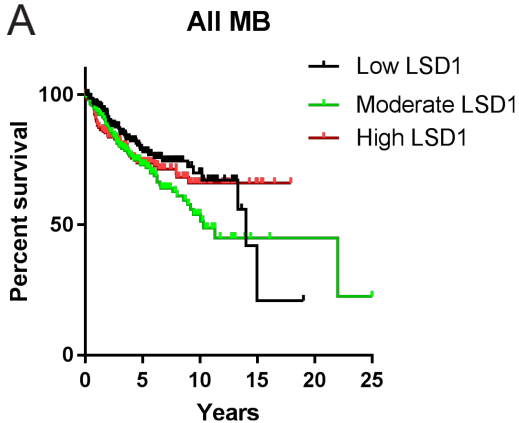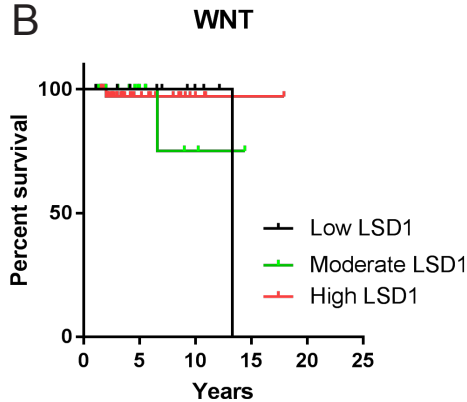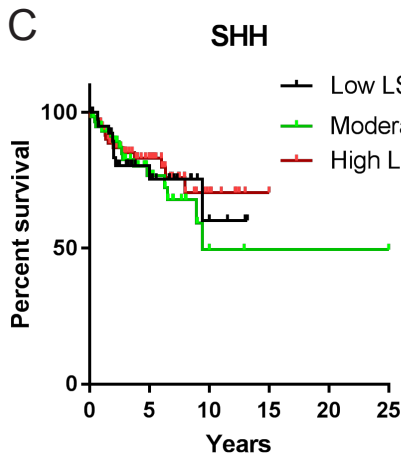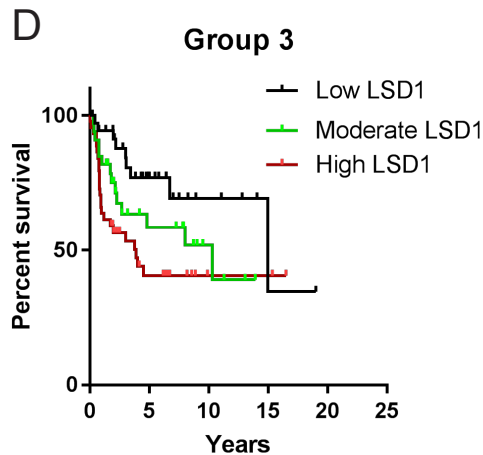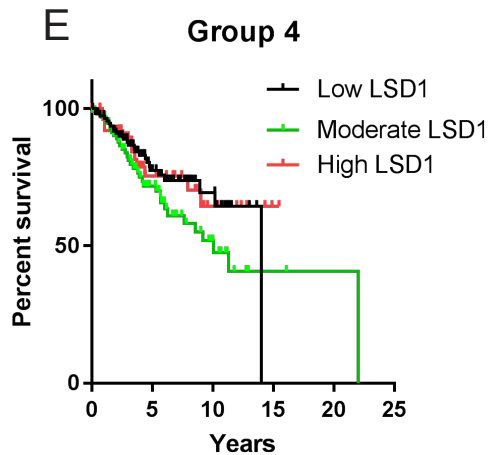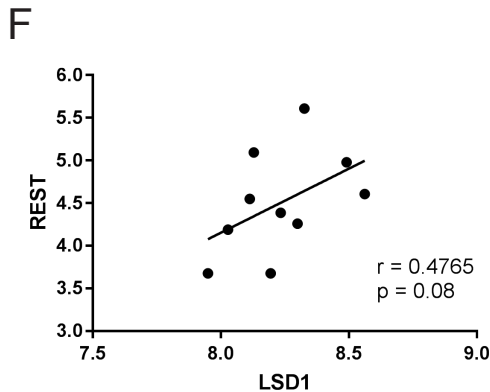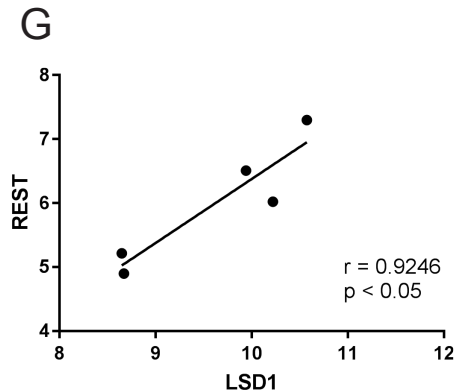

Supplement: Supplementary file 1 — A-E, Patient samples were stratified into low (n = 207), moderate (n = 210), and high (n = 213) LSD1 expression based on Z-score expression and survival curves were compared. A, Across all MBs, high and low LSD1 expression group survival curves were insignificant in Gehan-Breslow-Wilcoxon test of early survival (p = 0.10) but moderate and low LSD1 expression survival curves were significant in Mantel-Cox test (p < 0.05). B-E, Across the MB subgroups, only Group 3 samples had significantly difference curves. Overall survival data from Group 3 tumors related to LSD1 expression. High vs. low LSD1 had significant Gehan-Breslow-Wilcoxon and Mantel-Cox tests (p < 0.05). Moderate vs. low LSD1 had a significant Gehan-Breslow-Wilcoxon test (p = 0.06). Moderate vs. high LSD1 was insignificant. F, Scatter plot of REST and LSD1 transcript correlation in SHH MB patients from dataset GSE37418 (n = 10; r = 0.48; p = 0.08). G, Scatter plot of REST and LSD1 transcript correlation in SHH MB patients from dataset GSE109401 (n = 5; r = 0.92; p < 0.05). (PDF 987 kb) [file 12964_2018_275_MOESM1_ESM.pdf]

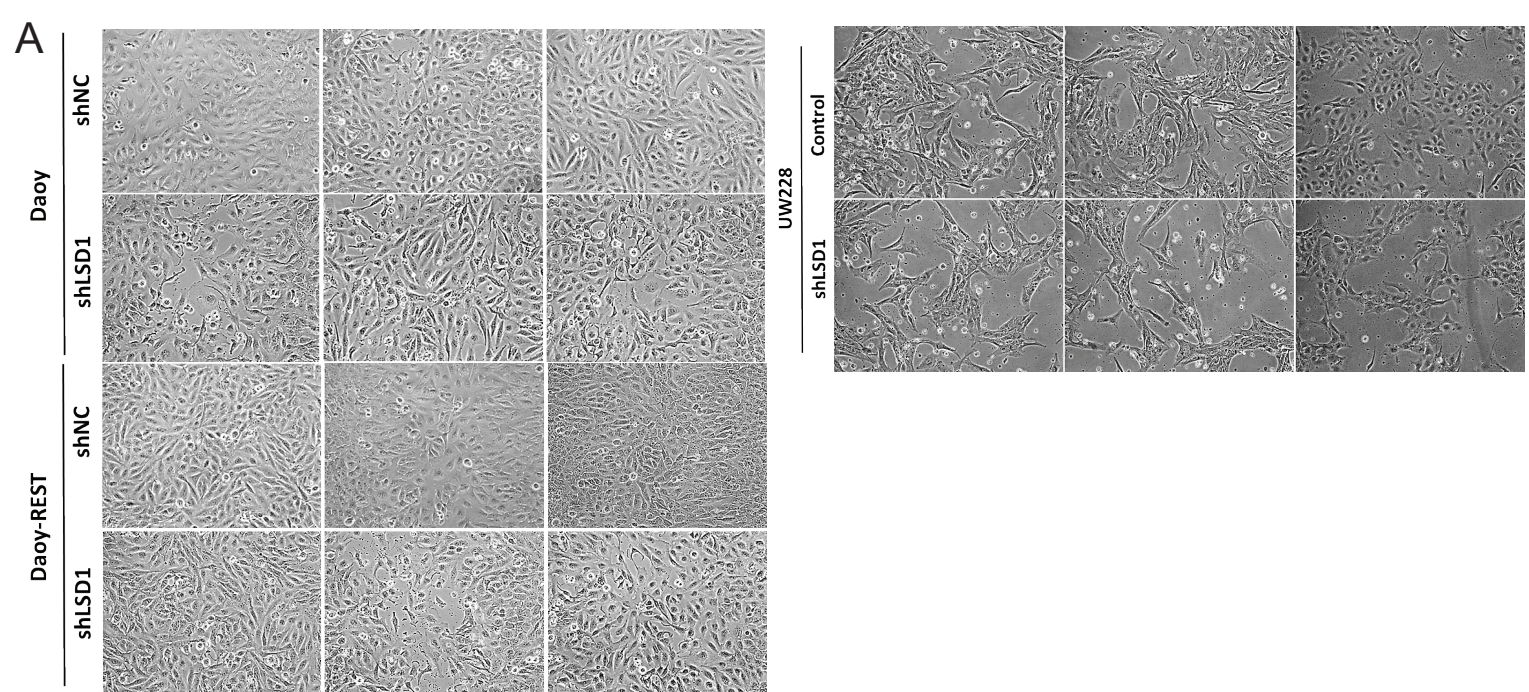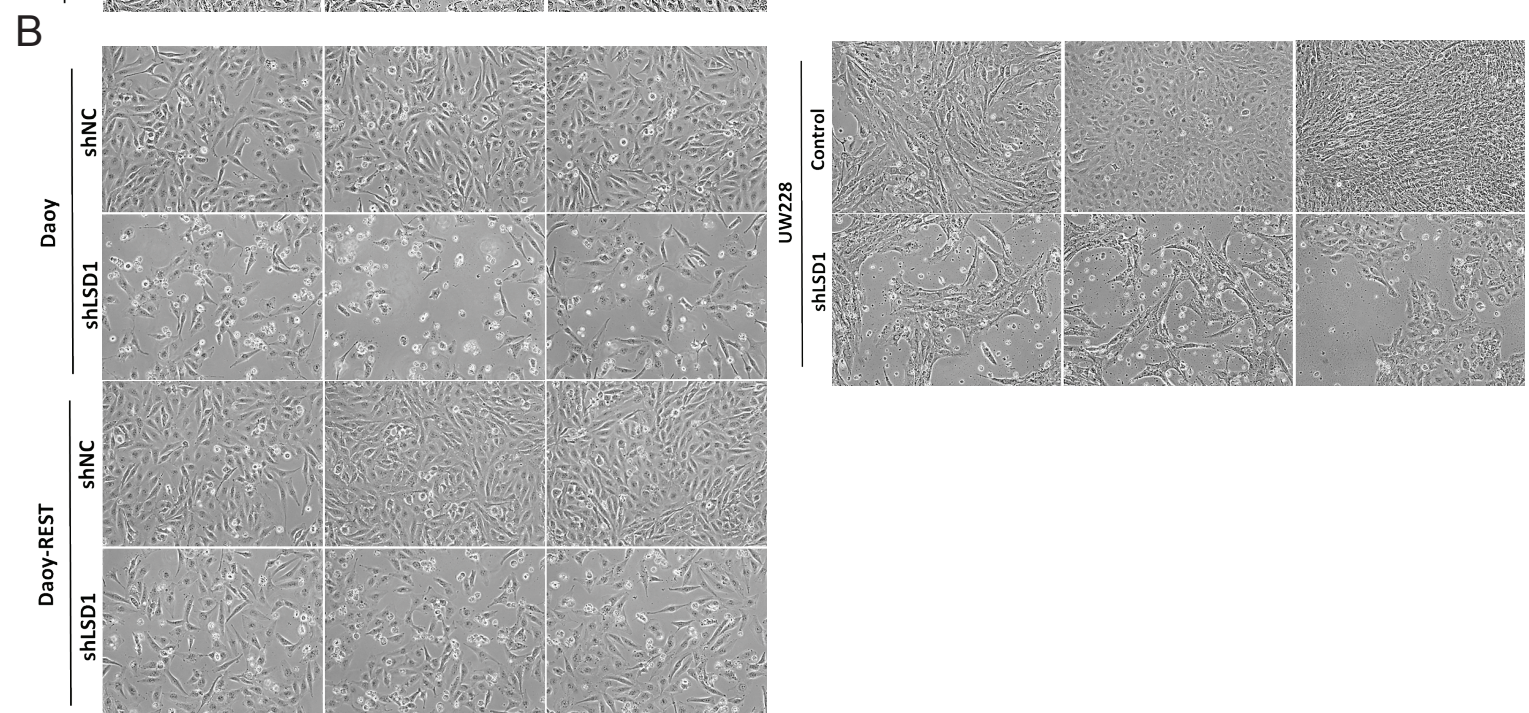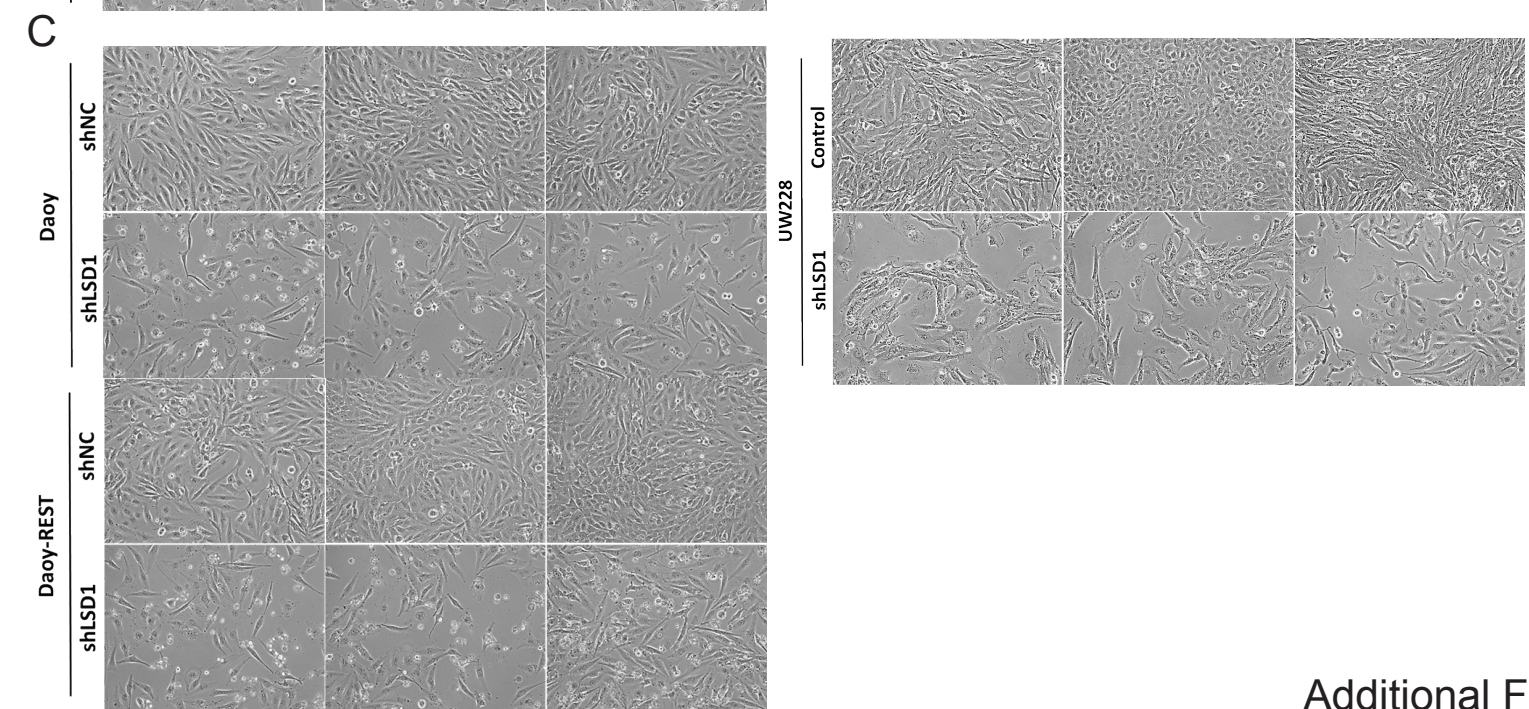

Supplement: Supplementary file 3 — Light microscopy images of Daoy, UW228, and Daoy-REST cells treated with control and shLSD1 at A, 24 h, B, 48 h, and C, 72 h. Experiments were completed in triplicate. (PDF 14333 kb) [file 12964_2018_275_MOESM3_ESM.pdf]

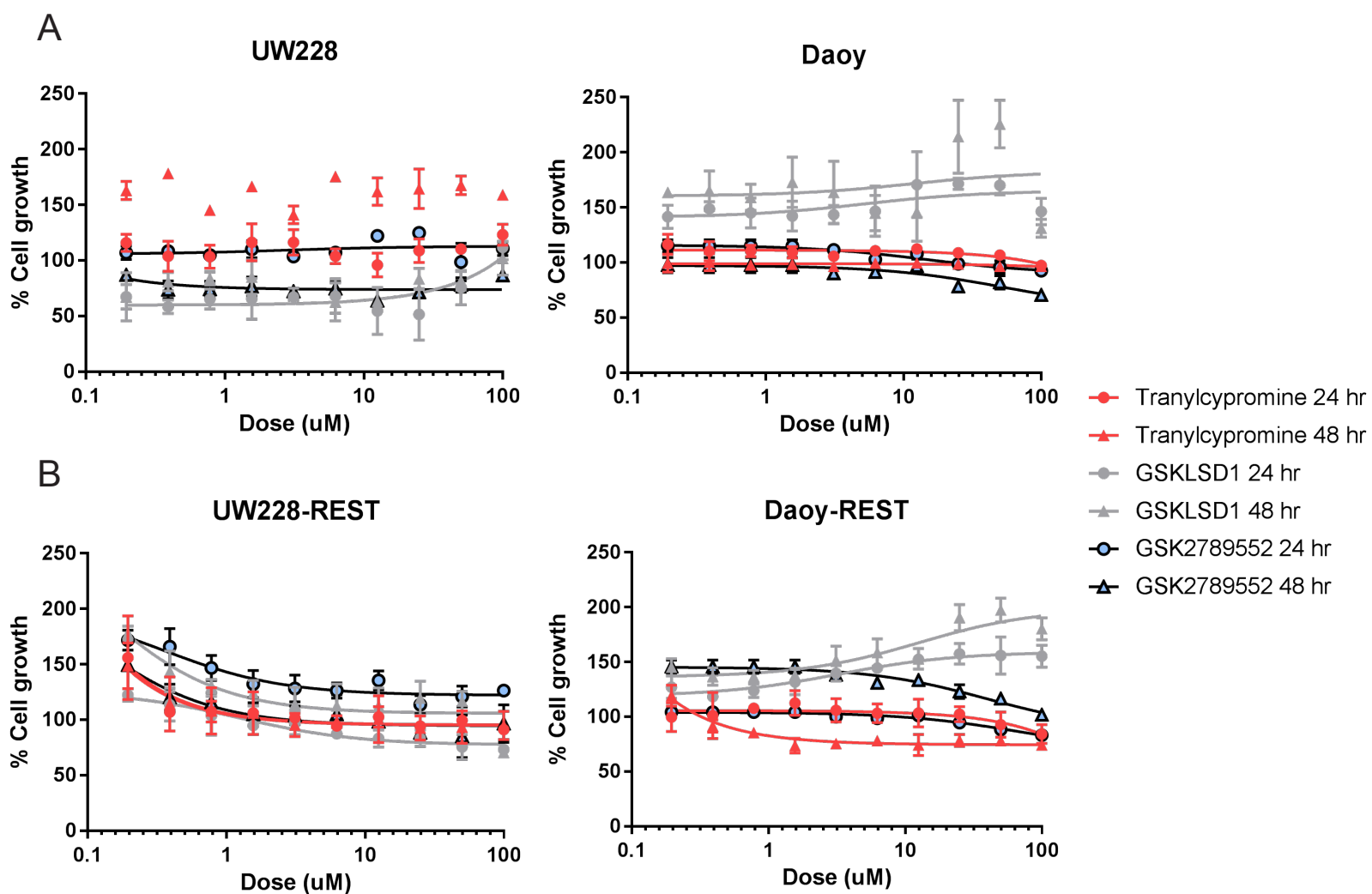

Supplement: Supplementary file 4 — A, MTT assay of 24 h and 48 h time points with three different LSD1 inhibitors (Tranylcypromine, GSKLSD1, and GSK2789552) in Daoy and UW228 cells showing no dose-response in response to up to 100 uM drug dosage. B, MTT assay of 24 h and 48 h timepoints with three different LSD1 inhibitors (Tranylcypromine, GSKLSD1, and GSK2789552) in isogenic high-REST counterparts, Daoy-REST and UW228-REST cells, showing no dose-response in response to up to 100 uM drug dosage. (PDF 1067 kb) [file 12964_2018_275_MOESM4_ESM.pdf]

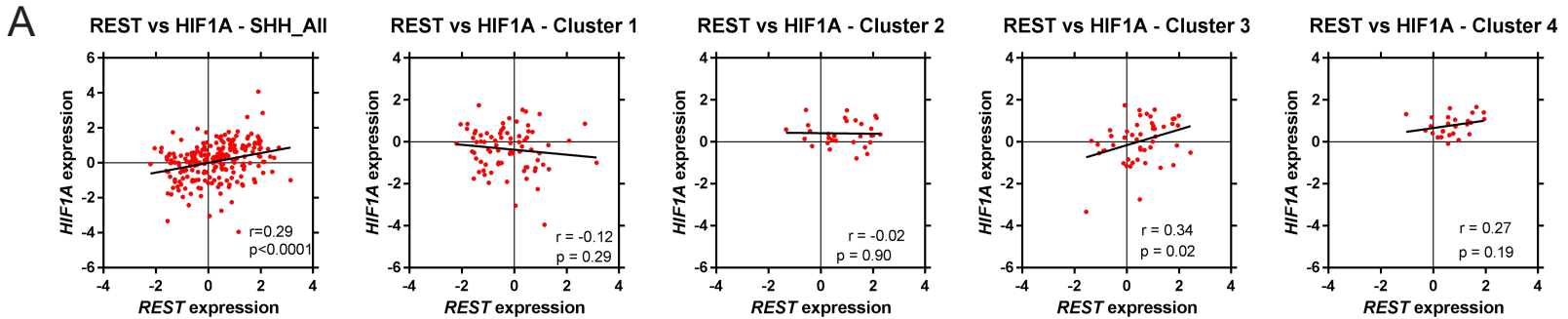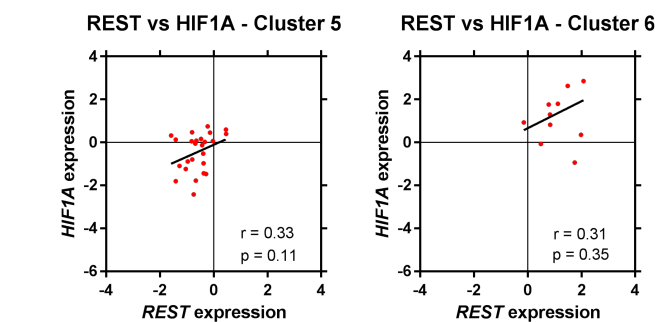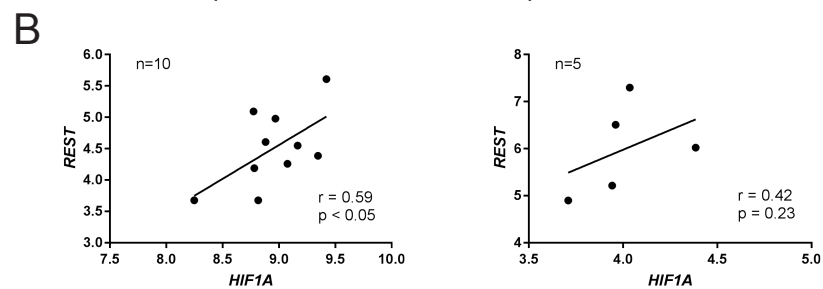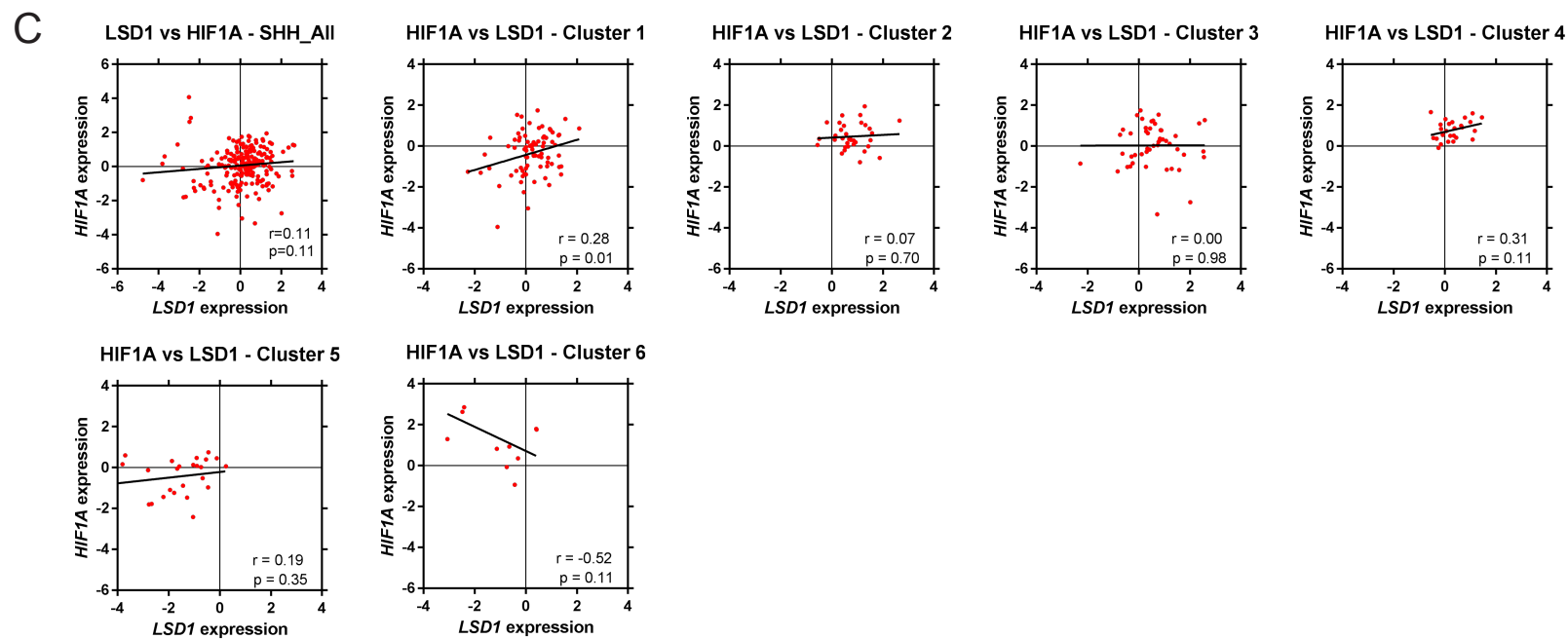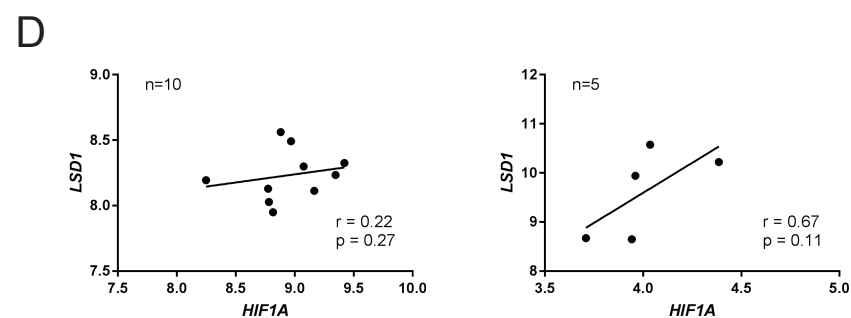

Supplement: Supplementary file 5 — A, Scatter plots of REST and HIF1A correlation across the whole SHH MB cohort and across each Cluster 1–6. Clusters 3 and 4 had majority of points located in the top right quadrant of the graph, indicating high expression of these transcripts, while Cluster 5 had lower left localization indicating lower expression levels. B, Scatter plots of REST and HIF1A transcript correlation in SHH MB patients from dataset GSE37418 (n = 10; r = 0.59; p < 0.05). G, Scatter plot of REST and HIF1A transcript correlation in SHH MB patients from dataset GSE109401 (n = 5; r = 0.42; p = 0.23). . C, Scatter plots of LSD1 and HIF1A correlation across the whole SHH MB cohort and across each Cluster 1–6. Clusters 2–4 had majority of points located in the top right quadrant of the graph, indicating high expression of these transcripts, while Cluster 5 had lower left localization indicating lower expression levels. D, Scatter plots of LSD1 and HIF1A transcript correlation in SHH MB patients from dataset GSE37418 (n = 10; r = 0.22; p = 0.27). G, Scatter plot of REST and HIF1A transcript correlation in SHH MB patients from dataset GSE109401 (n = 5; r = 0.67; p = 0.11). (Cluster 1 n = 78; Cluster 2 n = 34; Cluster 3 n = 49; Cluster 4 n = 26; Cluster 5 n = 25; Cluster 6 n = 11). (PDF 1382 kb) [file 12964_2018_275_MOESM5_ESM.pdf]
