# Supplementary material for: Pharmacological inhibition of LSD1 activity blocks REST-dependent medulloblastoma cell migration
Source: Cell Commun Signal. 2018 Sep 18;16:60. doi: 10.1186/s12964-018-0275-5 (PMC6145331; doi:10.1186/s12964-018-0275-5)
Supplement: Supplementary file 2 — A, Listed genes used for clustering analysis in the order of top-down pictured in Fig. 1C. B, Box plots of LSD1, REST, and USP7 expression (*p < 0.05;**p < 0.01; ***p < 0.001;****p < 0.0001). C, Scatter plots of REST and LSD1 correlation across the whole SHH MB cohort and across each Cluster 1–6 (Cluster 1 n = 78; Cluster 2 n = 34; Cluster 3 n = 49; Cluster 4 n = 26; Cluster 5 n = 25; Cluster 6 n = 11). D, Scatter plots of USP7 and LSD1 correlation across the whole cohort and across each Cluster 1–6. E, Kaplan-Meier survival curve of all Clusters 1–6. (PDF 1450 kb) [file 12964_2018_275_MOESM2_ESM.pdf]

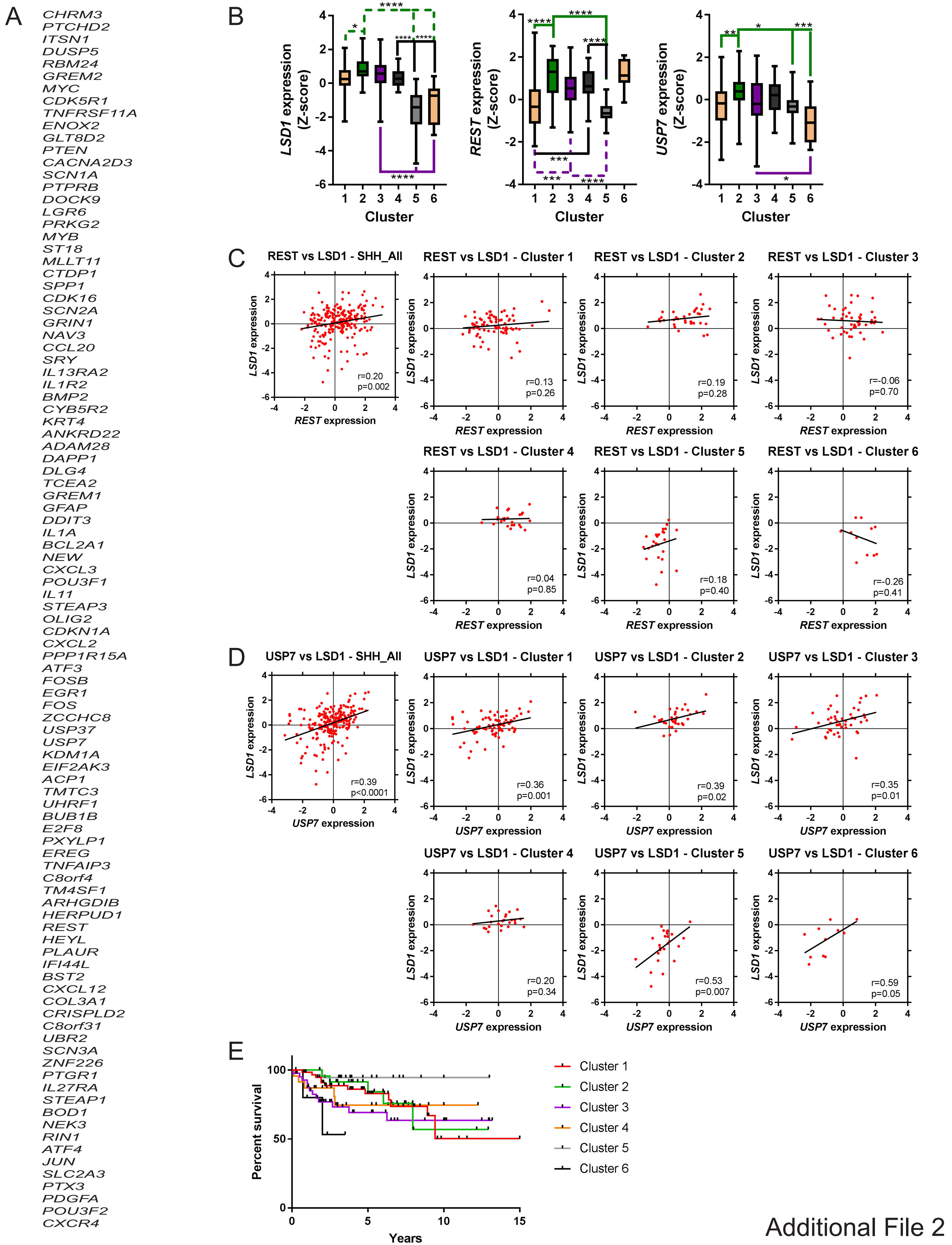

Additional File 2
